# Supplementary material for: parallelMCMCcombine: An R Package for Bayesian Methods for Big Data and Analytics
Source: PLoS One. 2014 Sep 26;9(9):e108425. doi: 10.1371/journal.pone.0108425 (PMC4178156; doi:10.1371/journal.pone.0108425)
Supplement: Appendix S1 — Remarks on kernels and bandwidth selection for semiparametric density product estimator method. (DOC) [file pone.0108425.s001.doc]

**Appendix S1**

**Remarks on kernels and bandwidth selection**

Given a kernel $K(\bullet)$ in $d$ dimensions, the expression $\frac{1}{h^{d}}K\left(\frac{\theta}{h}\right)$ in formulas for nonparametric and parametric estimators can be replaced by $\left|H\right|^{-1/2}K\left(H^{-1/2}\theta\right)$, where $H$ is the bandwidth $d\times d$matrix which is symmetric and positive definite. In this case, for the semiparametric estimator, in the formula for the weights , the matrix $h^{2}I_{d}$ will be replaced by $H$. Note that the default choices for the arguments **bandw** and **anneal** in the R package function **semiparamDPE()** are equivalent to setting the bandwidth *h* as it is defined in the semiparametric density product estimator algorithm of Neiswanger et al. [1], which is $h=t^{(-1/(4+d))},\text{ }t=1,...,T$, where *T* is the number of samples.

When using the semiparametric density product estimator method, often the choice of $H$is crucial in the estimation. In particular, if the bandwidth is a fixed diagonal matrix $H=\text{diag}\left(h_{1}^{2},h_{2}^{2},...,h_{d}^{2}\right)$, the smoothing parameters $h_{i}$ may be chosen according to Silverman’s rule of thumb

$h_{i}=\left(\frac{4}{d+2}\right)^{1/(d+4)}T^{(-1/(d+4))}\sigma_{i}$, (1)

where $\sigma_{i}$ is the standard deviation of the *i*-th scalar component of $\theta$, and *T* is the total number of samples (see Silverman [2], Wand and Jones [3,4] and Duong and Hazelton [5]). This option is included as an example in the **semiparamDPE()** function of the R package.

**References**

1. Neiswanger W, Wang C, Xing E (2014) Asymptotically exact, embarrassingly parallel MCMC. arXiv:1311.4780v2.
2. Silverman BW (1986) Density Estimation for Statistics and Data Analysis. London: Chapman and Hall/CRC Press.
3. Wand MP, Jones MC (1993) [Comparison of smoothing parameterizations in bivariate kernel density estimation](http://www.jstor.org/stable/2290332). J Am Stat Assoc 88: 520-528.
4. Wand MP, Jones MC (1995) Kernel Smoothing. London: Chapman and Hall/CRC Press.
5. Duong T, Hazelton ML (2003) Plug-in bandwidth matrices for bivariate kernel density estimation. J Nonparametric Statistics 15: 17-30.
